# Transcatheter Mitral Valve Replacement: Clinical Challenges, Evolving Indications, and Future Directions

**DOI:** 10.1016/j.shj.2026.101125

**Published:** 2026-09-07

**Authors:** Guido Ascione, Sebastian Ludwig, Augustin Coisne, Philipp von Stein, Nicolo Piazza, Firas Zahr, Vlasis Ninios, D. Scott Lim, David V. Daniels, Charanjit S. Rihal, Torsten P. Vahl, Thomas Modine, Juan F. Granada

**Affiliations:** a Clinical Trials Center, Cardiovascular Research Foundation, New York, New York, USA; b Department of Cardiology, University Heart & Vascular Center Hamburg, University Medical Center Hamburg-Eppendorf, Hamburg, Germany; c Department of Clinical Physiology and Echocardiography—Heart Valve Clinic, CHU Lille, Lille, France; d Department of Cardiology, Heart Center, Faculty of Medicine, University of Cologne, Cologne, Germany; e Department of Interventional Cardiology, McGill University Health Centre, Montreal, Quebec, Canada; f Knight Cardiovascular Institute, Oregon Health and Science University, Portland, Oregon, USA; g Cardiology Department, Interbalkan Medical Center, Thessaloniki, Greece; h Interventional Cardiology, University of British Columbia, Vancouver, British Columbia, Canada; i Interventional Cardiology, Sutter Health California Pacific Medical Center, San Francisco, California, USA; j Department of Cardiovascular Medicine, Mayo Clinic, Rochester, Minnesota, USA; k Columbia University Medical Center, New York Presbyterian Hospital, New York, New York, USA; l Medico-Surgical Department, Haut-Lévêque Cardiological Hospital; Bordeaux University Hospital, Pessac, France

**Keywords:** Mitral regurgitation, Transcatheter mitral valve replacement, TMVR

## Abstract

Mitral regurgitation is a highly prevalent valvular heart disease and, when hemodynamically significant, is associated with adverse outcomes. Despite advances in surgical techniques and catheter-based edge-to-edge repair, a large number of patients remain untreated due to high surgical risk or anatomical limitations. Transcatheter mitral valve replacement (TMVR) has emerged as a promising therapeutic option for these patients. Over the past 15 years, the TMVR field has substantially progressed, with multiple devices advancing to human clinical evaluation. This review aims to provide an overview of the key milestones in TMVR development, patient selection pathways, and the transition from transapical to transseptal delivery. We further summarize the available clinical outcomes across different platforms and discuss remaining knowledge gaps, as well as future directions for this evolving field.

## Highlights

• TMVR provides predictable MR elimination across complex anatomies.
• TMVR adoption has been limited by high screening failure rates.
• Screening failure reasons are evolving with device innovation and increasing understanding of optimal patient selection.
• Transseptal TMVR has narrowed the procedural-risk gap with M-TEER.
• Future TMVR adoption will require safer devices, broader anatomical eligibility, and proven durability.

## Graphical Abstract

The transcatheter mitral valve replacement development journey. MAC, mitral annular calcification; MR, mitral regurgitation; M-TEER, mitral transcatheter edge-to-edge repair; TMVR, transcatheter mitral valve replacement.

**Figure:**
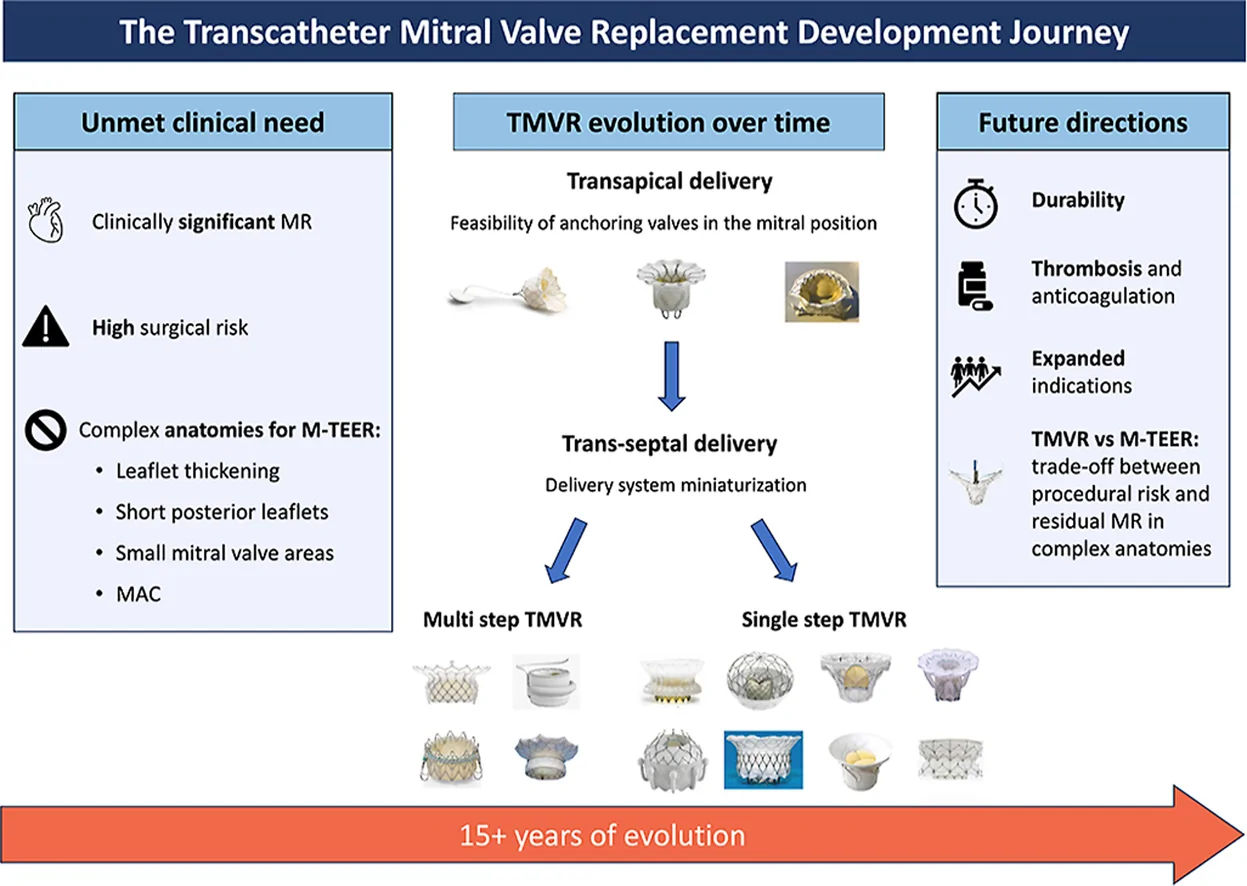

## Introduction

Mitral regurgitation (MR) is a highly prevalent valvular heart disease and, when hemodynamically significant, it is associated with increased mortality and health care costs.^1^ While surgery remains the standard of care, especially in the context of primary MR,^2^ a large number of MR patients are rejected from surgery due to their prohibitive risk, leaving a therapeutic gap.^3^ Mitral transcatheter edge-to-edge repair (M-TEER) has filled this gap with excellent procedural safety and broad availability.^4^ Yet many patients are ineligible for M-TEER because of the complexity and variability of mitral valve anatomy. Leaflet thickening, short posterior leaflets, clefts, wide jets, small mitral valve areas, and mitral annular calcification (MAC) limit the efficacy and wider clinical use of M-TEER, as they are associated with a higher risk of residual MR and iatrogenic mitral stenosis.^5^^,^^6^ In these cases, transcatheter mitral valve replacement (TMVR) offers a promising alternative, with the potential to provide more reproducible MR reduction. However, TMVR adoption has been limited by a high screening failure rate due to specific anatomical challenges, including the risk of left ventricular outflow tract (LVOT) obstruction, thereby slowing the introduction of these technologies into clinical practice.^7^

Over the last decade, several TMVR devices have been developed and reached clinical evaluation. Early TMVR technologies used the transapical (TA) route, given its direct access to the mitral valve. Nevertheless, this more invasive strategy carries an increased risk of morbidity and mortality.^8^ For these reasons, current development has shifted toward a fully transseptal (TS) approach. This review aims to provide a comprehensive summary of the status of the TMVR field, highlighting the lessons learned from early clinical experiences and the main challenges that need to be addressed in order to fully establish transcatheter replacement as a reliable therapeutic option for mitral disease in routine clinical practice.

## Methods

For each device, the most up-to-date available evidence was identified through a structured search of peer-reviewed publications (PubMed), company websites, ClinicalTrials.gov, and presentations from major structural heart meetings up to April 2026. When multiple data sources were available for the same device or cohort, more recent and complete data sets were prioritized over earlier reports.

## TMVR Position Within the Mitral Valve Disease Therapeutic Framework

According to the most recent European Guidelines for the Management of Valvular Heart Disease,^2^ surgical repair is considered the treatment of choice in primary MR when surgical risk is acceptable, with M-TEER recommended as an alternative in high-risk patients with suitable anatomy (Class IIa). For secondary MR, M-TEER holds a Class I recommendation for patients with a ventricular phenotype remaining symptomatic despite optimal medical therapy, provided anatomical and clinical criteria of success are met, while surgery may be preferred in the atrial phenotype (Class IIa) and in patients requiring concomitant coronary artery bypass grafting. To date, the only formal indication for TMVR is degenerative mitral stenosis with extensive MAC (Class IIb); the guidelines additionally acknowledge TMVR, without a formal recommendation, as an alternative to surgery in high-risk primary MR patients in whom M-TEER is anatomically unfeasible (i.e., severe MAC with or without concomitant stenosis, mitral valve area < 3 cm^2^, short posterior leaflets (< 5 mm), calcifications in the grasping zone, multiple jets, deep regurgitant clefts).^9^

## First-Generation TMVR Technologies: Design Considerations

In the early days of TMVR, secondary MR was thought to be the most appealing and immediate clinical target. Thus, device design and development were guided by the assumption that patients would typically present with large ventricles, noncalcified annuli, and low LV ejection fraction.^10^^,^^11^ As a result, most first-generation TMVR technologies relied on large valve designs that were difficult to downsize into smaller and flexible delivery catheters, therefore favoring the TA approach as the primary access route.

Several important lessons were learned from first-generation TA TMVR platforms that are no longer under development. Notable examples include the Tiara (Neovasc Inc., Richmond, BC, Canada) and Fortis (Edwards Lifesciences, Irvine, CA, USA) systems.^12^^,^^13^ Both technologies took advantage of the TA approach to clip and secure the transcatheter valve within the native mitral valve apparatus, using the leaflets as the primary anchoring mechanism. Despite high technical success rates,^14^^,^^15^ these programs were discontinued due to device-related complications including dislodgment, thrombosis, and LV dysfunction.^16^^,^^17^

First-generation TMVR technologies marked an important milestone by demonstrating successful delivery and anchoring in noncalcific valvular tissue. At the same time, they unveiled the challenges involved in platform development, independent of the delivery route. The marked heterogeneity of device designs over the course of TMVR evolution reflects the underlying anatomic complexity and the diverse pathophysiologic substrates these technologies aim to address.

## Transition to Transseptal TMVR Delivery

The first successful transfemoral implantation of a dedicated TMVR device was performed in Copenhagen in 2012 with the first-generation CardiAQ valve (CardiAQ Valve Technologies, Irvine, CA),^18^ using a nonsteerable delivery system mechanically guided by a snare and an arteriovenous loop. Besides this first single attempt, most of the devices developed in the following 5 years were engineered to be implanted transapically, with numerous first-in-human implantations performed through 2020.^13^^,^^19^, ^20^, ^21^, ^22^, ^23^, ^24^, ^25^ (Figure 1). In this early experience, outcomes were impacted by a high occurrence of bleeding and prolonged hospital stays, ultimately reducing the potential procedural advantages of TMVR over surgery in high-risk populations.^26^^,^^27^ Consequently, while some companies focused on catheter design modifications and profile reduction to enable TS delivery, others that designed their valve delivery mechanism around the TA access route failed in this transition and eventually discontinued their TMVR programs (i.e., Tiara and Fortis).^28^

**Figure 1:**
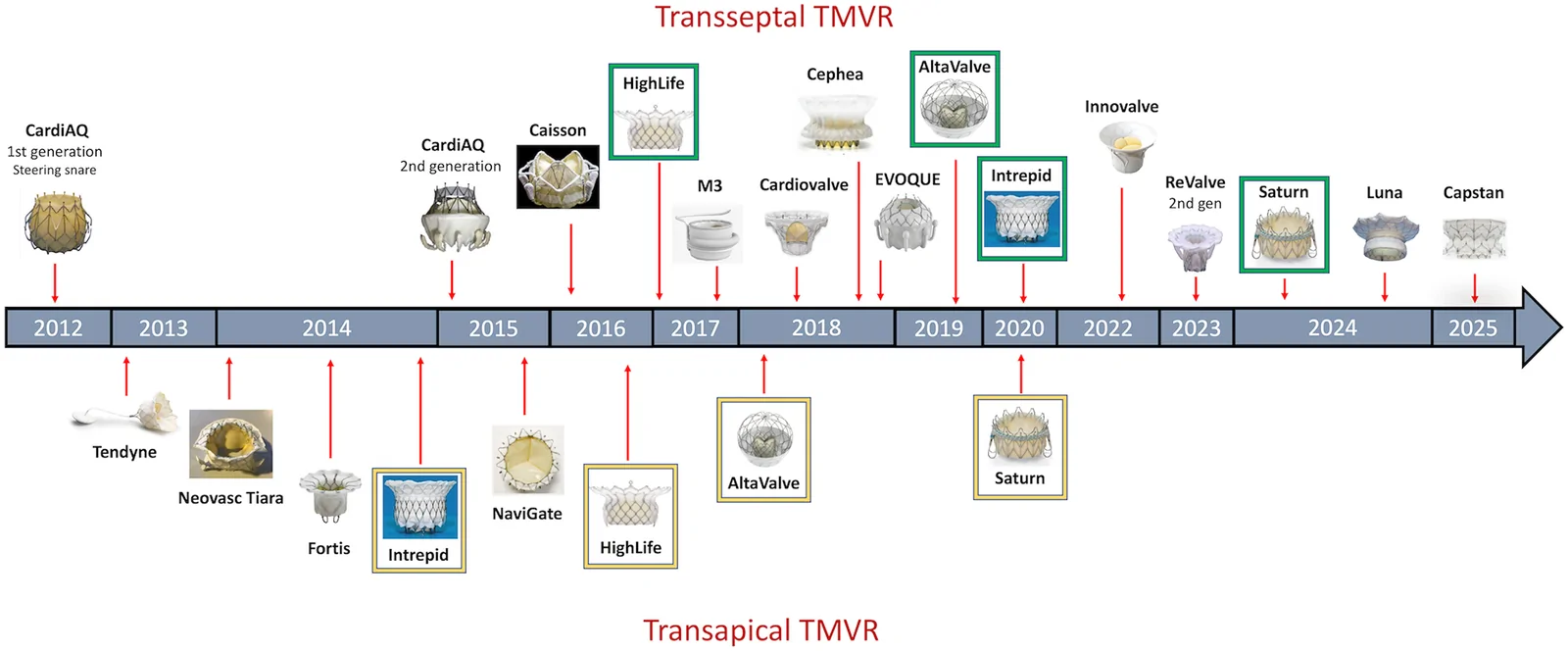
**Timeline of first-in-human implantation of different TMVR devices.** Valves that transitioned from an initial transapical approach to a subsequent transseptal one are highlighted in squares. Abbreviation: TMVR, transcatheter mitral valve replacement.

Delivery system miniaturization was made possible by separating the different steps of valve delivery and implantation, leading to the development of multistep TMVR systems. This approach rapidly enabled a reduction in catheter size by decoupling the anchoring mechanism (implanted during the first step of the procedure) from the valve itself (implanted during the second step). The first-in-human use of TS TMVR systems, performed between 2016 and 2018 (Figure 1), primarily relied on multistep technologies (e.g., HighLife [HighLife Medical, France] and SAPIEN M3 [Edwards Lifesciences, Irvine, CA, USA]). Although technically challenging, this strategy was accompanied by a progressive advancement in implantation experience and in the understanding of patient selection and fixation strategies. The field continued to evolve with the introduction of single-step TMVR technologies, with the goal of procedural simplification.^28^ The first single-step TMVR implantations were performed in 2018 (Cardiovalve [Venus Medtech, Israel] and Cephea [Abbott Vascular, Santa Clara, CA, USA], Figure 1).

The transition from a TA to a TS route of delivery has been associated with a substantial improvement in patients’ outcomes. As an example, in the Intrepid valve program (Medtronic Inc, Minneapolis, MN, USA), despite a slightly higher procedural success with the TA approach^29^ (98.4% vs. 93.9% after TS delivery), complication rates were consistently lower when the valve was delivered transseptally.^30^ Also, 30-day mortality decreased from 13.1% to 0%, the 1-year incidence of disabling stroke decreased from 3.6% to 0%, 1-year mortality from 27.3% to 6.7%, and the rate of heart failure–related rehospitalization from 26.1% to 6.7%. The only exception was the incidence of major bleeding, which remained high (27% in the TA cohort vs. 31% in the TS cohort at 1 year), likely reflecting the impact of post-TMVR anticoagulation in this frail and clinically complex population.

## TMVR Design and Anatomical Mismatch

The Achilles’ heel of TMVR has been its high screen failure rate, which occurred in up to 83.1% of patients treated with early-generation devices.^31^ Reasons for screen failure are multifactorial and related to the strict inclusion criteria of early feasibility studies that were primarily designed to assess device performance rather than long-term patient outcomes. In the early days of TMVR development, the majority of exclusions were due to anatomical reasons, most commonly (1) an annular size outside device ranges, (2) a high risk of LVOT obstruction, (3) small ventricular cavities with short papillary muscle distance, (4) severe MAC, and (5) low septal height. In the CHOICE-MI (CHoice of OptImal transCatheter trEatment for Mitral Insufficiency) registry, including 746 patients who were screened for TMVR at 26 centers from 2014 to 2021, anatomical ineligibility was the most common reason for screening failure (79% of the cases), with out-of-range annular dimension and risk of LVOT obstruction accounting for roughly two-thirds of the exclusions.^32^

Most of the programs have increased the number of available valve sizes and are now able to accommodate a greater number of annular dimensions referred to screening committees.^33^ Also, as operators gain experience with TMVR technologies, more complex anatomies are now being included. Consequently, a progressive shift in screening failure reasons has been observed across different platforms. Today, patient exclusion is primarily due to clinical factors: frailty, limited life expectancy, severe LV systolic dysfunction (with concern for post-implant afterload mismatch), severe pulmonary hypertension, right ventricular dysfunction, severe tricuspid regurgitation, end-stage kidney disease, or inability to take oral anticoagulation (OAC).^11^ As an example, in the Cephea valve’s early program, screen failure was mainly due to the existence of a single valve size, with annular–prosthesis mismatch accounting for 54% of exclusions. As multiple sizes became available, annulus-driven exclusions dropped to less than one-third, with the remainder split between risk of LVOT obstruction, septal-height constraints, and clinical profile.^34^

Screening failure reasons substantially differ according to MR etiology. In another analysis from the CHOICE-MI registry, secondary MR patients were mostly excluded because of their clinical characteristics (frailty, comorbidities, limited life expectancy), with a minor role played by annular areas that were too large for the available valve size or concerns about afterload mismatch. Conversely, in patients with primary MR, screening failure was often due to anatomical limitations.^35^ This likely reflects the fact that first-generation TMVR technologies were primarily designed for secondary MR and therefore continue to face major challenges in patients with primary MR, particularly related to smaller ventricular size and the presence of MAC.^33^

Venous access, per se, is rarely a cause of screening failure with current TS devices. However, delivery system size correlates with major vascular complications and clinically relevant iatrogenic atrial septal defects, reinforcing the importance of profile reduction even if access is technically feasible.^11^

Another often underappreciated reason for screening failure is septal height, which influences device steerability inside the left atrium. Systems that use shorter valves (e.g., Saturn Valve, InnovHeart, Milan, Italy) naturally demand less septal height because the smaller ventricular profile reduces the risk of interaction with papillary muscles or LV walls; others (e.g., Cephea Valve) have added height-gain controls to their delivery system to reach the optimal trajectory.

Also, multiple anatomical exclusions often coexist (e.g., MAC is usually associated with small annuli and often a narrow LVOT), creating clusters of patients that may be particularly hard to treat.^11^^,^^35^ With commercial approval of TMVR devices, however, the pool of eligible patients is expected to broaden, thanks to increasing operator experience and the possibility of overcoming the stringent inclusion/exclusion criteria of clinical trials.^11^

## TMVR and LVOT Obstruction Risk

Despite a progressive decline in anatomical unsuitability for TMVR, LVOT obstruction still remains a major challenge, responsible for up to one-third of screening failures with latest iteration devices.^34^ LVOT obstruction after TMVR is multifactorial, depending on both patient anatomy and valve design. On the anatomical side, many characteristics modulate the shape and size of the neo-LVOT, including the aortomitral angle, interventricular septal thickness, anterior mitral leaflet length, ventricular size, and papillary muscle position. On the device side, frame depth, frame shape, and skirt geometry have a direct role in defining the risk of LVOT obstruction. Even a well-sized prosthesis can obstruct the outflow tract if the frame sits too deep or shifts toward the septum during release. Moreover, some platforms (e.g., Saturn, HighLife, and SAPIEN M3), because of their subvalvular anchoring mechanisms, achieve a predictable “nominal” shape and size, which may allow for more reliable preprocedural prediction of neo-LVOT. In contrast, devices that anchor at the annular level may be underexpanded or fail to assume a consistent orientation due to variations in tilt or implantation depth, resulting in less reliable neo-LVOT estimations.

Newer TMVR platforms have converged on flatter, shorter, more tubular, and more open-cell geometries to reduce ventricular footprint and preserve outflow area, especially in small LVOT phenotypes.^10^ As an example, AltaValve (4C Medical Technologies, Maple Grove, MN) has been designed to primarily anchor at the atrium, minimizing ventricular intrusion.^36^ Innovalve (Edwards Lifesciences, Irvine, CA) is held in position by subannular arms that twist around native chordae, thereby drawing the anterior mitral leaflet away from the outflow tract.^37^ Cephea Valve employs a short ventricular disk with dynamic motion that, with each contraction, tends to move away from the septum, limiting outflow encroachment.^38^ Saturn features a low-profile frame and its subannular segments achieve some degree of anterior leaflet stabilization, therefore keeping the LVOT clear.^39^

Several adjunctive interventional tools are emerging and promise to play an important role in increasing the eligibility of patients with more complex anatomies. Two main techniques have been developed to modify the anterior mitral leaflet: the LAMPOON (Laceration of the Anterior Mitral Leaflet to Prevent Left Ventricular Outflow Tract Obstruction) technique^40^ and the BATMAN (Balloon Assisted Translocation of the Mitral Anterior Leaflet to prevent Left Ventricular Outflow Obstruction) technique.^41^ LAMPOON functions by creating a controlled midline incision in the anterior mitral leaflet, which then splays apart after TMVR deployment, thereby uncovering transcatheter heart valve cells that would otherwise be obstructed. Importantly, the chordal attachments to the papillary muscles are preserved, maintaining ventricular function and directing the incised leaflet halves outward in a safe manner.^42^ BATMAN, conversely, consists of valve deployment through a balloon-assisted perforation of the anterior mitral leaflet, ideally resulting in posterior translocation of the leaflet and related subvalvular apparatus, therefore clearing the LVOT.^41^ Among the techniques targeting the septum, both alcohol septal ablation (ASA)^43^ and SESAME (Septal Scoring Along Midline Endocardium)^44^ have been proven to be able to increase LVOT area. ASA consists of injecting dehydrated ethanol into a target septal perforator artery branching off the left anterior descending artery, therefore causing a localized infarction in the basal septum. Conversely, SESAME produces a transcatheter myotomy that immediately reduces LVOT gradient and eventually splays further over 30 days.^42^ Each of these techniques has its own pitfalls (i.e., uncontrolled balloon dilatation/electrosurgery laceration of the anterior leaflet with extension of the tear into the aortomitral curtain with the BATMAN and LAMPOON techniques, complete heart block or ventricular septal defect with ASA and SESAME, greater technical complexity of electrosurgical approaches compared with ASA), but the progressive accumulation of experience, and—eventually—the introduction of dedicated transcatheter electrosurgery devices are expected to broaden the adoption of these techniques among interventionalists performing TMVR.^42^ Nevertheless, further evidence is needed to better define the optimal strategies and valve designs for the prevention and management of LVOT obstruction during TMVR, as well as to establish the safety and appropriate target population for each available adjunctive interventional tool.

## TMVR and MAC

Patients with MAC have limited treatment options. Surgery is frequently considered high risk and may be complicated by atrioventricular groove rupture, circumflex artery injury, embolic events, and significant paravalvular leak.^45^ M-TEER is technically challenging because extensive calcification restricts leaflet mobility and reduces the available grasping area, increasing the likelihood of incomplete leaflet coaptation, iatrogenic mitral stenosis, and residual MR.

The MAC-related severe distortion of annular and often subvalvular anatomy also poses major challenges for TMVR, as it complicates device anchoring and increases procedural risk. Early approaches such as valve-in-MAC, involving implantation of balloon-expandable valves within the calcified annulus, are conceptually straightforward but technically unpredictable, with risks of paravalvular leak, device migration, and incomplete sealing, and reported 30-day mortality rates as high as 20.3%.^46^ For these reasons, dedicated TMVR platforms have attracted increasing interest as a means to overcome the intrinsic limitations of valve-in-MAC. The Tendyne prosthesis (Abbott Vascular, Santa Clara, CA, USA) was the first system successfully implanted in patients with MAC, demonstrating encouraging early outcomes in terms of technical success, perioperative complication rates, and short-term hemodynamic performance.^47^^,^^48^ Similarly, the Intrepid valve is currently being evaluated in this population within a dedicated arm of the APOLLO (Transcatheter Mitral Valve Replacement With the Medtronic Intrepid™ TMVR System in Patients With Severe Symptomatic Mitral Regurgitation) trial.^49^ Overall, TMVR represents a promising therapeutic option for patients with severe MAC and complex mixed mitral valve disease. Data from the CHOICE-MI registry suggest that, although moderate or severe MAC is associated with higher procedural complication rates, survival, MR reduction, and functional improvement are comparable to those observed in patients without significant calcification.^50^ However, success in this setting depends on meticulous preprocedural planning, advanced imaging, experienced heart teams, and continued technological refinement.^51^

## Current TMVR Landscape

In almost 15 years of TMVR experience, many different devices have been progressively designed and tested in clinical studies. An overview of the most current state of these devices, with a focus on their anchoring mechanism and early clinical outcomes, is provided in Tables 1 and 2.

**Table 1:** Overview of contemporary and historical TMVR devices that reached first-in-human implantation∗

	AltaValve^52^	Caisson^53^	Capstan^54^	Cardiovalve^55^	Cephea^56^	Eos^57^	Fortis^58^	HighLife^23^	Innovalve^59^
Sheath size	29 F	31 F	32 F	32 F	33 F	27 F	42 F	30 F	32 F
Valve	Bovine pericardial leaflets	Porcine pericardial leaflets	NA	Bovine pericardial leaflets	Porcine pericardial leaflets	Bovine pericardial leaflets	Bovine pericardial leaflets	Bovine pericardial leaflets	Bovine pericardial leaflets
Available sizes	Valve: 27 mm Annular ring: 40, 46, and 54 mm Cage: patient-specific	36 mm, 42 mm version A (smaller AP distance) and B (bigger AP distance)	40 mm	Medium (valve 27 mm, annulus diameter up to 45 mm), large (valve 29 mm, annulus diameter up to 50 mm), X-large (valve 29 mm, annulus diameter up to 55 mm)	28, 32, 36, and 40 mm	44 mm, 48 mm	Valve: 29 mm	31 mm (standard valve) Large annulus valve (increases annular range to 53 mm)	One valve size, atrial flange available in 2 sizes (54 mm, 59 mm)
Fixation location	Supraannular	Subannular	Annular	Subannular	Annular	Annular/subannular	Subannular	Subannular	Subannular
Delivery route	Transseptal	Transseptal	Transseptal	Transseptal	Transseptal	Transseptal	Transapical	Transaortic retrograde and transseptal	Transseptal
Number of steps	Single step	Multistep	Single step	Single step	Single step	Single step	Single step	Multistep	Single step
Anchoring mechanism	Atrial wall oversizing	4 subannular anchoring feet, 3 atrial holding features	2 rows of ventricular cleats	Multiple ventricular legs that secure the valve to native leaflets	Atrial and ventricular discs	Atrial shoulders, nitinol frame, and ventricular anchors	2 paddles to anchor to mitral leaflets, atrial flange	Ring	6 arms extend radially from valve body and engage subvalvular apparatus

	Intrepid^49^	Luna^76^	M3^92^	NaviGate^93^	Saturn^75^	Revalve^85^	Tendyne^65^	Tiara^12^
Delivery system size	29 F	28 F	29 F	32 F	29 F	NA	34 F	32 F
Valve	Bovine pericardial valve	Bovine pericardial valve	Bovine pericardial valve	Equine pericardial valve	Bovine pericardial valve	Bovine pericardial valve	Porcine pericardial leaflets	Bovine pericardial valve
Available sizes	42 and 48 mm	Single size (28 mm) covering annular diameter up to 50 mm	29 mm	36, 40, 44, 48, 52 mm	28 and 31 mm	32 mm	Single valve size, multiple outer frame sizes	35 and 40 mm
Fixation location	Annular	Subannular	Subannular	Annular/subannular	Subannular	Annular/subannular	Annular/apical	Subannular
Delivery route	Transseptal	Transseptal	Transseptal	Transapical	Transseptal	Transseptal	Transapical	Transapical
Number of steps	Single step	Multistep	Multistep	Single step	Multistep	Single step	Single step	Single step
Anchoring mechanism	Outer stent anchors grasp annular tissue using a cork effect	Subannular ring	Docking system that encircles mitral chordae and leaflets	Annular winglets and ventricular graspers that engage leaflets and subvalvular apparatus	Two annular half-circles positioned behind the native leaflets, interlocked with the prosthetic valve via connecting arms.	2 clips (capture leaflets), 2 stabilizers (annulus anchoring), and an atrial flange	Apical tether and pad	Ventricular anchors secure the valve onto the fibrous trigones and posterior shelf of the annulus

Abbreviations: AP, anteroposterior; F, French; NA, not available; TMVR, transcatheter mitral valve replacement.

∗ Here we present the latest available valve iteration of each program, which may differ from the version tested in first-in-human and Early Feasibility Studies.

**Table 2:** Early clinical outcomes across platforms

Clinical development stage∗	AltaValve^52^^,^^60^	Caisson^53^	Capstan^54^	Cardiovalve^55^	Cephea^61^^,^^62^	Eos^57^	Fortis^16^	HighLife^63^	Innovalve^59^^,^^64^
	EFS (30 patients: 13 implanted TA, 17 TS)	FIH/EFS (29 patients)	FIH (2 patients)	FIH (15 patients)	EFS (24 patients)	EFS (60 patients)	FIH (13 patients)	EFS (30 patients)	EFS (35 patients)
In-hospital outcomes									
Technical success	97% (n = 29)	76% (n = 22)	100% (n = 2)	87% (n = 13)	96% (n = 23)	100% (n = 60)	77% (n = 10)	90% (n = 27)	94% (n = 33)
LVOT obstruction	0%	0%	0%	0%	8% (n = 2)	2% (n = 1)	0%	0%	0%
Major access site complication	0%	NA	0%	NA	NA	0%	NA	7% (n = 2)	0%
30-d outcomes									
All-cause mortality	10% (n = 3); 3% in the TS cohort	14% (n = 4)	0%	NA	4% (n = 1)	5% (n = 3)	38% (n = 5)	10% (n = 3)	3% (n = 1)
≥ Major bleeding	NA	NA	0%	NA	12% (n = 3)	28% (n = 17)	15% (n = 2)	17% (n = 5)	14.3% (n = 5)
MR ≥ 2	0%	NA	0%	NA	0%	2% (n = 1)	0%	0%	PVL: 36% (n = 10) Transvalvular: 11% (n = 3)
1-y outcomes									
All-cause mortality	24% (n = 7); 7% in the TS cohort	21% (n = 6)	NA	NA	29% (n = 7)	20% (n = 12)	46% (n = 6)	17% (n = 5)	24% (n = 7)
HFH	NA	21% (n = 6)	NA	NA	37.5% (n = 9)	27.1% (n = 16)	NA	23% (n = 7)	34.5% (n = 10)
≥ Major bleeding	NA	34% (n = 10)	NA	NA	12% (n = 3)	30% (n = 18)	NA	23% (n = 7)	34% (n = 10)
MR ≥ 2	5% (n = 1)	7% (n = 2)	NA	NA	0%	0%	0%	0%	PVL: 15% (n = 2) Transvalvular: 0%
Stroke	0%	10% (n = 3)	NA	NA	4.2% (n = 1)	1.7% (n = 1)	NA	3% (n = 1)	6.9% (n = 2)
Valve thrombosis	3% (n = 1)	NA	NA	NA	NA	NA	NA	NA	NA
MV reintervention	0%	31% (n = 9)	NA	NA	0%	5.1% (n = 3)	NA	3% (n = 1)	6.9% (n = 2)

Clinical development stage∗	Intrepid^30^	Luna^76^	M3^73^	NaviGate^22^	Saturn^39^^,^^75^	Revalve^85^	Tendyne^48^^,^^66^^,^^94^	Tiara^15^
	EFS (33 patients)	FIH (1 patient)	Pivotal (299 patients)	FIH (1 patient)	FIH/EFS (6 patients, 5 implanted TA and 1 TS)	FIH (3 patients)	EFS: 191 patients RCT roll-in arm: 100 patients	FIH/EFS/Pivotal (71 patients)
In-hospital outcomes								
Technical success	94% (n = 31)	100% (n = 1)	96% (n = 287)	100% (n = 1)	100% (n = 6)	100% (n = 3)	95% (n = 279)	93% (n = 66)
LVOT obstruction	3% (n = 1)	0%	0%	0%	0%	0%	NA	0%
Major access site complication	24% (n = 8)	0%	3% (n = 9)	0%	0%	0%	NA	1% (n = 1)
30-d outcomes								
All-cause mortality	0%	0%	0.7% (n = 2)	0%	0%	0%	9% (n = 26)	11% (n = 8)
≥ Major bleeding	27% (n = 9)	0%	8.7% (n = 26)	0%	0%	0%	22% (n = 63)	NA
MR ≥ 2	0%	0%	4.3% (n = 12)	0%	0%	0%	1% (n = 3)	3% (n = 1)
1-y outcomes								
All-cause mortality	7% (n = 2)	NA	13.9% (n = 40)	NA	0%	0%	29% (n = 85)	NA
HFH	6.7% (n = 2)	0%	16.7% (n = 47)	0%	0%	0%	EFS: 29.3% (n = 56)† Roll-in: NA	NA
≥ Major bleeding	31% (n = 10)	NA	18.5% (n = 52)	NA	0%	0%	29% (n = 84)	NA
MR ≥ 2	0%	NA	4.3% (n = 10)	NA	0%	0%	4% (n = 7)	0%
Stroke	0%	NA	5.5% (n = 11)	NA	NA	NA	EFS: 3.7% (n = 7)† Roll-in: 5.4% (n = 5)	NA
Valve thrombosis	3.4% (n = 1)	NA	6.7% (n = 19)	NA	NA	NA	EFS: NA Roll-in: 2.3% (n = 2)	NA
MV reintervention	3.4% (n = 1)	NA	6.4% (n = 18)	NA	NA	NA	EFS: 0%† Roll-in: 2.2% (n = 2)	NA

Abbreviations: EFS, Early Feasibility Study; FIH, First-in-Human; HFH, heart failure hospitalization; LVOT, left ventricular outflow tract; MR, mitral regurgitation; MV, mitral valve; NA, not available; PVL, perivalvular leaks; RCT, randomized controlled trial; TA, transapical; TS, transseptal.

∗ This row indicates the clinical development stage from which the data in this table are derived, corresponding to the most advanced stage with publicly available results. Some programs (Caisson, Eos, Fortis, Navigate, and Tiara) have been discontinued.

† Two-year outcomes.

### The Tendyne Transapical System

The Tendyne platform consists of a valve sewn within 2 self-expanding nitinol stents. The inner stent is circular while the outer stent is D-shaped to conform to the native mitral annulus. An atrial flange provides sealing and fixation at the atrial level, whereas a LV apical tethering system with an external apical pad reduces paravalvular insufficiency and facilitates apical closure.^65^ Over the years, extensive clinical experience has been gained with this system. Both the Global Feasibility Study and the Expanded Clinical Study demonstrated its durable efficacy up to 3 years, with freedom from mitral reoperation of 96.9% and mild or less residual MR in all patients.^66^ These results led to Conformité Européenne (CE) Mark approval in 2020. More recently, the US SUMMIT (Clinical Trial to Evaluate the Safety and Effectiveness of Using the Tendyne Transcatheter Mitral Valve System for the Treatment of Symptomatic Mitral Regurgitation) Pivotal trial confirmed favorable outcomes, reporting procedural success of 97% and mild or less MR in 96.5% of patients at 12 months in the roll-in cohort, and procedural success of 98%, 30-day mortality of 6.8%, and mild or less MR in all patients at 30 days in the MAC cohort.^48^ Based on these findings, the device has recently received Food and Drug Administration (FDA) approval for patients with severe MAC who are deemed too high risk for surgery and unsuitable candidates for M-TEER.

## Multistep TMVR Systems

### HighLife

The HighLife TMVR device is a 2-component system. The valve prosthesis, composed of glutaraldehyde-fixed bovine pericardial leaflets mounted on a nitinol frame, is delivered transseptally. Anchoring is achieved through interaction with a dedicated subannular implant, which is positioned beforehand via retrograde aortic access.^67^ After a few cases performed transapically,^23^ this program transitioned to a fully percutaneous delivery; early feasibility study results have been encouraging, with a technical success rate of 90%, a 1-year mortality of 17%, and no patient demonstrating more than mild MR at 12 months.^63^ Similar promising results have been confirmed in a recent report on the first 35 patients reaching 3-year follow-up (all-cause mortality 42.9%, mild-or-less MR in all treated patients).^68^ The HighLife TMVR device recently received CE Mark approval,^69^ thereby increasing the portfolio of options for patients that are deemed unsuitable for surgery and M-TEER.

### SAPIEN M3

The SAPIEN M3 TMVR system comprises 2 sequentially deployed components: a self-expanding nitinol docking system and a balloon-expandable SAPIEN 3 valve adapted for mitral implantation.^70^ The US EFS demonstrated the safety, feasibility, and efficacy of this device in high–surgical-risk patients with severe symptomatic MR. Conducted across 4 US centers, the trial enrolled 35 patients and achieved a technical success rate of 88.6%. At 30 days, all-cause mortality was 2.9%, with 87.9% of patients presenting with none/trace MR and 12.1% with mild MR.^71^ Based on these promising results, the SAPIEN M3 received CE Mark approval in April 2025, becoming the world’s first commercially available transfemoral TMVR system.^72^ The ENCIRCLE (SAPIEN M3 System Transcatheter Mitral Valve Replacement Via Transseptal Access) trial (NCT04153292) is currently ongoing, with a dedicated cohort for patients with failed M-TEER and severe MAC, and its 1-year results have been recently published. In a sample of 287 patients who underwent valve implantation, the primary endpoint (nonhierarchical composite of all-cause mortality and heart failure hospitalization at 1 year) occurred in 25.2% of patients, significantly lower than the prespecified performance goal of 45%. Moreover, the mitral valve reintervention rate was 6.4%, and 95.7% of the cohort was free from more-than-mild MR at 1-year follow-up.^73^ The SAPIEN M3 device recently received FDA approval, becoming the first TS TMVR platform commercially available in the United States.^74^ Edwards has also announced a postmarket registry following commercialization.^72^

### Saturn

The Saturn valve consists of 2 interlocking components that form a unified structure. The native mitral leaflets are secured in the subannular space by 2 annular segments, which are mechanically connected to the central valve through a series of linking arms, ensuring stable fixation.^39^ Originally designed for TA delivery, the device showed encouraging 1-year results in 5 patients who were implanted transapically, with no deaths and complete elimination of MR.^39^ The platform has since been adapted to a multistep TS delivery system, with the first-in-human TS implantation performed in May 2024.^25^ The CASSINI-EU EFS (Reduction of Mitral Regurgitation With the SATURN Trans-Septal Transcatheter Mitral Valve Replacement (TMVR) System in Patients With Severe Symptomatic Mitral Regurgitation Early Feasibility Study) is currently underway to evaluate the safety and performance of this device in its TS configuration.^75^

### Luna

The Luna TMVR device (Tioga Cardiovascular Inc, Los Gatos, CA, USA) is a 2-step TS system with dedicated delivery platforms for the anchor (subannular nitinol ring with polymer covering) and the valve (made of bovine pericardial leaflets, with a large atrial brim and short outflow). The first-in-human implantation was performed in October 2024 and resulted in complete MR abolition maintained through 30-day follow-up.^76^ A multicenter first-in-human study, designed to enroll up to 30 patients, is currently ongoing.^77^

## Single-Step TMVR Systems

### EVOQUE/Eos

The EVOQUE mitral valve (Edwards Lifesciences, Irvine, CA, USA) features a self-expanding nitinol frame carrying bovine pericardial leaflets, an intra-annular sealing skirt, and ventricular anchoring elements.^78^ The first-in-human implantation was performed in 2018, and an initial experience including 14 patients (8 under compassionate use and 6 within an early feasibility study) reported encouraging outcomes, with procedural success of 93%, one death, and no cases of more than mild MR at 30 days.^78^ While this first-generation design was further developed for the tricuspid position, a second-generation valve, called Eos, was engineered to fit in a 27F delivery system, the smallest to date among all TMVR platforms, and evaluated in the MISCEND (Edwards EVOQUE Eos Mitral Valve Replacement: Investigation of Safety and Performance After Mitral Valve Replacement With Transcatheter Device) study. In 60 patients, the device achieved 100% procedural success, with 30-day and 1-year mortality rates of 5 and 20%, respectively, and 100% freedom from more than mild MR at 1 year.^57^ However, following an unusually high incidence of LV pseudoaneurysms (10% at 1 year) occurring at the basal portion of the LV and therefore related to the interaction with the valve’s anchors, the Eos mitral program was recently discontinued.^57^

### Cardiovalve

The Cardiovalve system is a self-expanding trileaflet bovine pericardial valve mounted within dual atrial and ventricular frames. It is delivered through a single-step TS procedure and is specifically engineered to maintain a low ventricular profile, thereby minimizing the risk of LVOT obstruction.^55^ The AHEAD (European Feasibility Study of the Cardiovalve Transfemoral Mitral Valve System) trial (NCT03339115) is currently underway to assess the safety and feasibility of this device. Early data from 15 patients demonstrated a technical success rate of 86.7%, with all treated patients exhibiting mild or less MR at hospital discharge. The longest follow-up reported to date extends to 5 years, showing no evidence of structural valve deterioration, no significant recurrent MR, and sustained improvement in clinical status.^55^ More recently, the delivery system has been completely redesigned to reduce screening failures and enable safe, coaxial deployment within the mitral valve apparatus. Early compassionate-use experience with the new system has been encouraging, with 100% technical success and no adverse events through 30-day follow-up.^79^

### Intrepid

The Intrepid TMVR system consists of a trileaflet bovine pericardial valve mounted within a self-expanding nitinol frame featuring a dual-stent architecture: a circular inner stent housing the valve and a conformable outer fixation ring that engages the native mitral annulus. A flexible brim is attached to the atrial end of the fixation ring, facilitating fluoroscopic and echocardiographic visualization during implantation.^80^ Originally designed for TA delivery,^29^ the system has since evolved into a fully transfemoral, single-step implantation platform. Thirty patients were enrolled in the US transfemoral EFS, demonstrating encouraging 2-year outcomes. Indeed, all-cause mortality was 16.8%, residual MR graded mild or less in all patients and no significant perivalvular leaks were recorded.^81^ Moreover, in 92 high-risk patients who underwent TA TMVR with the first generation of the valve, 5-year mortality and heart failure hospitalization rates were 66.7 and 55.4%, but only 1 patient required a mitral valve reintervention, residual MR was mild or less in all patients, and there were no cases of severe hemodynamic valve deterioration at 5 years.^82^ The APOLLO (NCT03242642) and APOLLO-EU (Transcatheter Mitral Valve Replacement With the Medtronic Intrepid™ TMVR System in Patients With Severe Symptomatic Mitral Regurgitation - Europe) (NCT05496998) pivotal trials, also including patients who underwent valve implantation with the new 29F transfemoral delivery system, are currently ongoing to validate these findings and to further define the patient populations most likely to benefit from this device.^49^

### AltaValve

The AltaValve system consists of a spherical self-expanding nitinol frame, intentionally oversized to the left atrium for anchoring, housing a trileaflet bovine pericardial valve. A fabric skirt is positioned at the base of the frame (annular ring) to minimize paravalvular leakage.^24^ Originally conceived for TA delivery, the valve has since been optimized for single-stage TS implantation via a dedicated 29F system.^36^ An ongoing early feasibility study has demonstrated encouraging outcomes. Among the first 30 enrolled patients (13 TA and 17 TS), 95% were free from more than mild MR at 1 year, and mortality rates at 30 days, 6 months, and 1 year were 10, 14, and 24%, respectively.^52^^,^^60^^,^^83^ Similarly favorable results have been reported in patients with severe MAC, where anchoring relies on a “transcatheter aortic valve replacement (TAVR)-like” mechanism of stent-based apposition against calcifications. In an initial series of 6 such patients, technical success was achieved in all cases, with one 30-day death and less than moderate MR in all survivors.^84^ The ATLAS (A Transseptal Left Atrial System for Treatment of Mitral Regurgitation) pivotal trial (NCT06465745) is currently enrolling in the United States and Europe to further evaluate the safety and efficacy of this device.

### Cephea

The Cephea valve is a single-step TMVR system characterized by a distinctive architecture comprising a ventricular disk, a central leaflet-carrying core, and an atrial disk. The device achieves fixation on the mitral annulus through axial compression forces, thereby avoiding the need for subvalvular anchors, pads, or tethers.^38^ The second-generation iteration incorporates a perivalvular leak–reduction skirt and open ventricular cells designed to mitigate the risk of LVOT obstruction. Early clinical experience in 31 patients demonstrated a high technical success rate (96%), 30-day mortality of 3%, and freedom from more than mild MR in all patients at 30 days.^61^ Similar results were reported in the cohort of 24 patients with available 1-year follow-up, with an all-cause mortality of 29.2% (but all deaths occurring in patients with a Society of Thoracic Surgeons score ≥8, chronic kidney disease stage ≥4, or pulmonary hypertension), and 97% of patients exhibiting no residual MR.^62^ A US pivotal trial is planned^56^ and will incorporate the third generation delivery system, featuring an additional knob that allows for height-depth adjustment at a wide range of septal crossing locations.^62^

### Innovalve

The Innovalve Transcatheter Mitral Valve Replacement System consists of an atrial skirt, a cylindrical frame carrying 3 bovine pericardial cusps, and 6 radially extending arms that interact with the subvalvular apparatus. During deployment, the arms are gradually rotated to engage the native mitral valve, ensuring secure anchoring and sealing, minimizing paravalvular leak, and displacing the native anterior leaflet away from the LVOT.^37^ Among the first 35 patients enrolled in the TWIST EFS (TVMR With the Innovalve System Trial-Early Feasibility Study), technical success was achieved in 94.3%, while mortality at 30 days and 1 year was 2.9 and 24.1%, respectively. All patients were free from more than mild MR at 1 year.^59^^,^^64^ In addition, significant LV reverse remodeling was observed, with a reduction in mean LV end-diastolic volume of 21 mL from baseline to 6 months, likely attributable to both the abolition of MR and the rotational maneuver of the arms that approximate the papillary muscles during implantation.^37^^,^^64^

## Future Directions

Early experience with dedicated TMVR systems has consistently shown near-complete MR elimination in eligible patients, accompanied by symptomatic improvement.^16^^,^^26^^,^^30^^,^^63^ Moreover, novel device designs continue to emerge, addressing some of the technical challenges identified to date (e.g., the ReValve technology (ReValve Solutions, Irvine, CA, USA), a single-step TS device that can be implanted through a low TS puncture (<4 cm),^85^^,^^86^ or the Capstan Medical valve (Capstan Medical, Santa Cruz, California, USA), the first TMVR system specifically designed for delivery via an integrated proprietary robotic arm^54^). The available clinical experience supports the core therapeutic promise of TMVR as a valid treatment option for MR, while also highlighting limitations that need to be addressed to enable broader adoption (Graphical abstract).

First, thrombosis and anticoagulation remain important challenges. TMVR prostheses are bulkier than M-TEER devices and reside in low-shear atrioventricular flow, predisposing to leaflet thickening and device thrombosis; accordingly, most programs, largely extrapolating recommendations from surgical bioprosthetic mitral valves, mandate OAC with vitamin K antagonists for at least 3 months after implantation.^78^^,^^87^^,^^88^ TMVR candidates, however, are frequently frail, which—together with large-bore access—helps explain the still nontrivial bleeding rates reported, including late events beyond the index hospitalization (see Table 2). Device-level solutions (more open frames, flow-friendly skirts) and patient-tailored antithrombotic strategies may help to mitigate this problem in the future. In addition, recent observational data suggest that, compared with vitamin K antagonists, direct oral anticoagulants may reduce bleeding complications in TMVR recipients without increasing thrombotic events, with the advantage of simplified anticoagulation management.^89^ However, these findings derive from nonrandomized studies with limited sample size and therefore should be interpreted with caution.

Second, durability must be demonstrated if TMVR is to expand into younger or lower-risk populations. Short-term follow-up is reassuring—multicenter Tendyne 3-year outcomes and 5-year results of TA Intrepid implantation show sustained MR abolition without structural valve deterioration in survivors^66^^,^^82^—but long-term (>5–10 years) durability remains unknown, with only sparse case-level data beyond that horizon.^90^ Encouragingly, when failure occurs, valve-in-TMVR rescue has been reported, suggesting a feasible lifecycle strategy analogous to TAVR.^91^

Whether transcatheter replacement will remain a complement for anatomies where an optimal M-TEER result is unlikely (e.g., severe MAC) or will compete with/replace M-TEER more broadly is an unresolved question. M-TEER’s safety profile—including the absence of a routine need for long-term OAC—sets a high bar^4^; yet the field’s shift from TA to TS approaches, smaller delivery systems, and better patient selection has begun to narrow the procedural-risk gap while preserving the TMVR’s hallmark of predictable and sustained MR elimination also in anatomies where repair underperforms. Defining the future position of TMVR within the treatment landscape of mitral valve disease will likely require direct comparisons with M-TEER, although a hypothetical randomized trial evaluating the 2 strategies would face several challenges. To ensure genuine clinical equipoise, enrollment would need to be limited to patients who are anatomically suitable for both therapies. Given the device-specific eligibility criteria currently required for TMVR, such a selected population may prove too small to support adequately powered studies. Furthermore, although the safety profile of TMVR has improved considerably with newer-generation devices and increasing operator experience, concerns regarding procedural complexity, device thrombosis, LVOT obstruction, vascular access, and the need for long-term anticoagulation may still reduce the willingness of both physicians and patients to accept randomization when M-TEER represents a well-established and less invasive therapeutic option.

As TMVR moves from feasibility to device-approval trials, success will hinge on carrying forward lessons learned on screening failure and procedural optimization and, equally, on defining the patients most likely to derive durable benefit from each device. Broader adoption will require safe, simple (potentially single step), and effective approaches that could even be performed under conscious sedation, analogous to TAVR. Ultimately, these advances will determine whether TMVR stays a niche complement to M-TEER and surgery or evolves into a co-equal pillar of MR therapy.

## Limitations

Several limitations of the present review should be acknowledged. First, due to its narrative nature, this work is inherently subject to the authors' interpretation and evidence weighting, which may introduce a degree of source selection bias. Second, although we tried to include the most up-to-date information available on each program, our literature search was influenced by the complexity of data gathering in this rapidly evolving field. Indeed, most evidence comes from conference presentations, few peer-reviewed data are available, and frequent device modifications during the early feasibility phase contribute to heterogeneity across the reported outcomes. Third, the devices discussed are in very different stages of clinical development; this review and its summary tables are therefore intended to describe individual program results rather than support direct device-to-device comparisons.

## Conclusions

TMVR has proven to be a safe and reproducible treatment option for mitral disease, with high procedural success in appropriately selected patients. Long-term outcome data are still pending and will be pivotal to guide the next device iterations. Given the wide anatomical heterogeneity of the mitral apparatus and LVOT, a single TMVR solution is unlikely to suffice. Instead, a diversified portfolio of complementary designs—spanning annular, subannular/docking, and supra-annular architectures with varying frame depths and LVOT-sparing features—will be required to address real-world variability and broaden eligibility without compromising safety.

## Funding

The authors have no funding to report.

## Disclosure Statement

S. Ludwig has received a grant from the German Heart Foundation (DHS) and 4C Medical; speaker honoraria from Abbott and Edwards Lifesciences; travel compensation from Edwards Lifesciences and Shockwave Medical; advisory fees from Bayer; and is a consultant for NVT. N. Piazza has served as a consultant for Medtronic; has received grants from Medtronic and St. Jude Medical; and has equity in CardiAQ and Highlife. F. Zahr has received grant support/research support from Edwards Lifesciences and Medtronic; and has received consultant fees/honoraria from Edwards Lifesciences and Medtronic. D.S. Lim is a consultant for Abbott, Corvention, Johnson & Johnson, LagunaTech, Medtronic, Venus, Valgen, and WL Gore; and has received research grants from Abbott Laboratories, Boston Scientific, Edwards Lifesciences, and Medtronic. D.V. Daniels is a consultant for Edwards Lifesciences. C.S. Rihal has received research grants from Edwards Lifesciences. T.P. Vahl has reported institutional funding to Columbia University Irving Medical Center from Boston Scientific, Edwards Lifesciences, JenaValve, and Medtronic and has received consulting fees from Abbott Vascular, 4C Medical, Innovheart, and Philips. T. Modine has received speaker or consulting fees from Abbott, Edwards Lifesciences, Medtronic, Microport, and GE. J.F. Granada has received institutional grant/research support from Abbott, Boston Scientific, Medtronic, and Edwards; and is the co-founder of Cephea Valve Technologies (Abbott). The other authors had no conflicts to declare.
